# Comparison of Two Types of Composite-Opaquer Combinations Masking Ability for Tooth Discoloration without Clinically Visible Opacity 

**DOI:** 10.30476/dentjods.2024.100117.2192

**Published:** 2025-06-01

**Authors:** Hossein Chalakinia, Farideh Darabi, Yasaman Sadeghi, Reza Tayefe Davalloo, Maryam Tavangar, Aidin Sooratgar

**Affiliations:** 1 Dept. of Restorative Dentistry, Mashhad University of Medical Sciences, Mashhad, Iran.; 2 Dept. of Restorative Dentistry, Dental School, Guilan University of Medical Science, Guilan, Iran.; 3 Dept. of Endodontics, Dental school, Tehran University of Medical Science, Tehran, Iran.

**Keywords:** Composite Resin, Meta-Color-Opaquer, Perceptual Masking, Tooth Discoloration

## Abstract

**Background:**

A few studies have examined the masking ability of the combined application of opaquer and resin composites on tooth discoloration.

**Purpose:**

This study investigated the combined application of opaquer-composite to mask discolored backgrounds by utilizing two opaquers and resin composites.

**Materials and Method:**

In this *in vitro* research, we used two composite resins, Vitalescence (Ultradent) and Gradia Direct (GC), and two opaquers: Pink opaque (Cosmedent) and Creative Color A2 (Cosmedent). Seventy-two specimens were made as disks in 8 groups (n=9), with 1.0mm and 0.5mm thicknesses. One surface of each disk was coated by one of the opaquers and cured. The 4mm-thick composite disks from both composite brands were also made as a reference, representing the inherent color of the material. Then, the composite disks from the opaquer-coated surface were placed on a C4 porcelain disk as a background to simulate a discolored tooth. Color measurement for all specimens was performed using a spectrophotometer device. The color difference of each test group and 4 mm thick disks of the same composite were measured using the formula CIELAB (ΔE * ab). Statistical analysis was performed by using ANOVA and Tukey's Post hoc tests.

**Results:**

Statistically significant differences were observed between the eight groups (*p*= 0.001). The thickness, composites, and opaquer variants significantly affected the ΔE*. The samples with 1mm thickness, the Vitalescence composite, and the Creative Color opaquer A2, had significantly lower ΔE* than the 0.5mm samples, the Gradia composite, and the Pink opaquer, respectively. The number of samples with desirable ΔE was significantly higher in the Vitalescence composite group than in the Gradia composite, and the Creative Color A2 opaquer had significantly more desirable ΔE than the Pink opaquer.

**Conclusion:**

Most results that achieved acceptable masking ability were obtained with combinations of Creative Color opaquer A2+ Vitalescence / Gradia composite in 0.5 or 1mm thicknesses.

## Introduction

Dental discoloration is one of the most important reasons for referring patients to dental clinics because it significantly impacts individuals' appearance and social relationships. Treating a discolored tooth is one of the most challenging issues in everyday dentistry. It requires proper diagnosis, a thorough understanding of etiology, and a precise plan to achieve a successful outcome [ 1 ].

Both endogenous and exogenous causes can cause discoloration. Pulpal necrosis, calcification, genetic and congenital diseases, and incomplete removal of endodontic materials are essential examples of endogenous causes. The discoloration induced by exogenous agents such as colored food and drinks, smoking, and inadequate oral hygiene are usually milder [ 2 - 3 ]. When discolored teeth do not show internal bleaching indications or internal bleaching is ineffective, direct composite veneers can be considered one of the conservative treatments for reproducing natural tooth color [ 4 - 6 ]. 

Resin Composites are inherently limited in the characteristic of opacification. Therefore, effective coating of dark colors is a great challenge. When dentin composites are used to mask the darkness, depending on the severity of discoloration, a high thickness of the restorative materials is usually required to completely mask the discoloration in the final restoration. This involves either removing a large portion of the dental tissue or accepting an over-contoured restoration [ 1 , 7 - 8 ]. According to the previous studies, at least 0.5mm- thick of opaque shade composite or 1.5mm thickness of final restoration with layering technique is needed to mask the C4 background color [ 9 ]. Miotti *et al*. [ 6 ] evaluated the ability of three resin composite systems (IPS Empress Direct, Charisma Diamond, and Filtek Z350 XT) in 1.5mm thicknesses to mask the C4 background by applying a layering technique. According to their results, only three combinations of Z350, which had 1mm dentin+0.5 mm enamel or body shade, could mask the background. In study of Perez *et al*. [ 10 ], 1.5mm-thick of two resin composites composed of 1 mm dentin and 0.5 mm enamel shade could not mask severely discolored substrates (C3, C4).

Ghorab *et al*. [ 11 ] showed that 0.5 mm thick of two opaque shade composites could mask the C4 background color. However, dentin or body shade composite should be used over it to modify the color of opaque shade composite.

Thus, to have minimum teeth preparation, using opaquers and tints is essential for proper coating of the discoloration . Opaquers contain varying amounts of opacifying metal oxides, such as titanium or aluminum oxide. Regardless of the type of opaquer, they should be used in very thin layers of 0.1 to 0.5mm. Some can mask a dark discoloration with a very thin coat (0.1mm); others may need two or more coats [ 1 , 4 , 13 ]. In addition, the color of the opaquer should be in harmony with the final color restoration [ 4 , 12 - 13 ]. After using the opaquer, a layer of a translucent composite should be used to remove the resulting opacity and produce a natural optical view [ 4 , 14 ].

Lehr *et al*. [ 15 ] evaluated the combined application of the opaquer and resin composite to mask discolored backgrounds (A3, A3.5, C2, C3, and C4). They reported acceptable results using 0.5-mm-thick body-shade resin composite combined with one or two coats of opaquer for the mildly discolored backgrounds (A3, A3.5, C2).

However, there are few studies about the combined application of opaquer and resin composite, most of which are case reports [ 1 , 7 , 12 - 16 ].

This study investigated the combined application of opaquer-composite to mask discolored backgrounds by utilizing two opaquers and resin composites.

The null hypothesis was that there was no difference between the two composites, their thicknesses, and the opaquers in the combined application of composite and opaquer to mask a discolored background. 

## Materials and Method

The pink opaquer was selected based on the manufacturer's claim that it is effective in masking gray discolorations and maintaining tooth vitality. The A2-shade opaquer was used according to previous studies' recommendations on the color harmony of opaquer with composite [ 12 - 13 , 16 ]. The studied materials are listed in Table 1. 

**Table 1:** Materials used in this study

Material	Manufacturer	Composition	Filler Size and Percentage	Lot No.
Vitalescence(A2 shade)	Ultradent	Bis-GMA, UDMA	Microhybrid	BDBZS
	Salt Lake City, Utah, USA	Silanated strontium borosilicate, Silanated silicon dioxide	0.7µm	
			71.5 % by wt.	
Gradia Direct (A2 shade)	GC	UDMA	Microhybrid	1705111
	Corporation	Flouro-alumino silicate glass, silica and prepolymerized filler	0.85 µm	
	Tokyo, Japan		75.0 % by wt.	
			64-65 % by v.	
Creative color	Cosmedent, Chicago, Illinois, USA	Bis-GMA	10-12 % wt.	160401A
Pink opaque		UDMA, 1,4-Butanediol dimethacrylate (85%), silicon dioxide filler (0.04µ),8% by volume, pigments (&lt;2%)		
Creative Color opaquer (A2 shade)	Cosmedent, Chicago, Illinois, USA	Bis-GMA	10-12 % wt.	153919C
		UDMA, 1,4-Butanediol dimethacrylate (85%), silicon dioxide filler (0.04µ), 8% by volume, pigments(up to 2%)		
Super Porcelain	Kuraray, Tokyo, Japan		Potassium-aluminosilicate glass, inorganic pigments	DUODD
EX-3 (C4B)				

Bisphenol A-glycidyl methacrylate (Bis-GMA), Urethane dimethacrylate(UDMA)

### Groups

The sample size was determined based on the study of Kim *et al*. [ 5 ], with 95% confidence and 90% test power, with nine composite disks in each group. One side of each disk was coated with one of the opaquers. In addition, disks with a thickness of 4 mm from each composite were prepared without opaquer to determine the inherent color of the composites. Composites and opaquers shade was A2 (Table 2). 

**Table 2:** Studied groups

Groups	Composite	Thickness	Opaquer
Numbers			
1	Vitalescence	0.5 mm	Pink opaque
2	Vitalescence	1.0 mm	Pink opaque
3	Vitalescence	0.5 mm	Creative opaquer A2
4	Vitalescence	1.0 mm	Creative opaquer A2
5	Vitalescence	4.0 mm	--
6	Gradia Direct	0.5 mm	Pink opaque
7	Gradia Direct	1.0 mm	Pink opaque
8	Gradia Direct	0.5 mm	Creative opaquer A2
9	Gradia Direct	1.0 mm	Creative opaquer A2
10	Gradia Direct	4.0 mm	--

### Preparing samples

**Figure 1:**
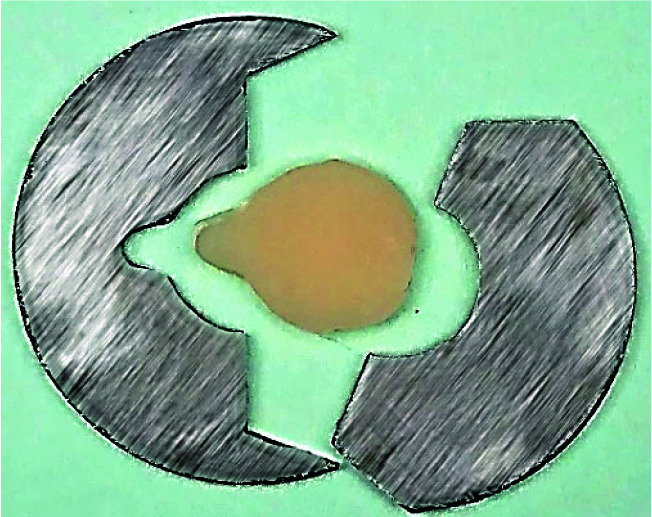
Split metal mold and composite disk

The split metal molds with 0.5 and 1 mm thicknesses and an 8 mm hole in the center were used. The molds were placed on a glass slab, and the holes were slightly overfilled with composite to ensure no voids remained. Another glass slab was placed on the top of the mold, and a weight of 1 kg was put on it for 10 seconds to exert pressure. The samples were polymerized from both sides for 40s using an LED light curing unit (Bluedent LED Smart, Bulgaria) at 800-1000 mW/cm2. The light intensity was monitored using a radiometer (RD-7, Ecel Ind. E Com. Ltda, Ribeirão Preto/São Paulo, Brazil). Then, the samples were placed in 37 °C distilled water for 24 hours to complete polymerization. The thickness of each disk was measured using a digital caliper (INSIZE, Stuttgart, Germany), and they were reduced to the desired thickness using abrasive discs. Both sides of the samples were polished for 20 s using medium, fine, and superfine aluminum oxide disks (Soflex, 3M ESPE, St. Paul, USA). One sample and the metal mold are shown in Figure 1. 

Two thin layers of each opaquer (about 0.2mm) were placed on one side of the composite disk using a small brush and then cured [ 12 ].

Porcelain C4 (Kuraray, Tokyo, Japan) was used to simulate the color of the discolored tooth [ 4 , 6 ]. For this, a porcelain C4 disk with a 4mm thickness and 8mm diameter was provided. A disk with the exact dimensions was made from wax and placed in refractory plaster to make the porcelain background. After the plaster setting, it was placed in a furnace to burn out the wax.

Then, the space was used as a mold to make the samples and heated according to the manufacturer's instructions. 

### Color measurement

A spectrophotometer (Color-Eye 7000 A; Gretag Macbeth, USA) was used to measure the color of the samples. The device was operated by an Institute for Color Science and Technology (Tehran, Iran) technician. Before analysis, the spectrophotometer was calibrated according to the manufacturer’s instructions. Each sample was placed on a C4 porcelain background and placed in a color-matching instrument. A glycerin optical fluid (Cargille Immersion Oil Type B, Cargille Laboratories, NJ) created optical contact between the disks and the background. The color of the 4-mm samples was measured on a neutral gray ceramic background (18%).

The color evaluation was performed using the parameter of CIE-L*a*b*, where L* is lightness, from zero (completely black) to 100 (completely white), a* is the green-red axis, and b* is the yellow-blue axis.

### Color Difference calculation

The difference between each sample on the C4 background and the inherent color of the material (∆E) was calculated as follows: 

ΔE*=(ΔL*)2+(Δa*)2+(Δb*)2(1/2)

Based on previous studies, if the ∆E* value is less than 3.3, the color difference is indistinguishable for the human eye [ 4 , 5 , 17 ]. This means the eye cannot detect the color difference between the composite-opaquer combination and the composite alone. 

The collected data were analyzed using version 21.0 of the SPSS software. The normal distribution of the data was evaluated by the Shapiro-Wilk test. The one-way ANOVA test was used to compare the ΔE between the groups. The Tukey post hoc test was used to compare differences between specific pairs of means between the groups.

Chi-Square or Fisher’s exact test was applied to compare the distinguishable changes (∆E lower or higher than 3.3). A *p* Value of less than 0.05 was considered statistically significant. 

## Results

The Shapiro-Wilk test revealed that data in the eight groups were normally distributed (*p*> 0.05). One-way ANOVA revealed a significant difference between the eight groups (*p*= 0.001). The lowest and highest ΔE were observed for Vitalescence (1mm) + Creative Color opaquer A2 and Gradia (0.5mm)+ Pink opaquer, respectively (Table 3). 

**Table 3:** Comparison of ΔE between the studied groups

	N	Mean	Std. Deviation	95% Confidence Interval for Mean		Minimum	Maximum	P*
				Lower	Upper			
Vitalescence 0.5mm+ Pink opaque	9	4.91	.82	4.27	5.54	3.25	5.91	.001
Vitalescence 1mm + Pink opaque	9	2.63	.31	2.39	2.87	2.21	3.30	
Gradia 0.5mm +Pink opaque	9	8.39	.33	8.13	8.64	7.73	8.93	
Gradia 1mm +Pink opaque	9	4.95	.42	4.63	5.27	4.40	5.47	
Gradia 1mm +Creative opaquer A2	9	1.22	.59	.76	1.67	.76	2.69	
Vitalescence 1mm+ Creative opaquer A2	9	1.34	.93	.63	2.06	.46	3.26	
Vitalescence 0.5mm+creative opaquer A2	9	.73	.33	.48	.98	.42	1.41	
Gradia 0.5mm +Creative opaquer A2	9	2.75	.53	2.35	3.15	1.66	3.60	
Total	72	3.36	2.49	2.78	3.95	.42	8.93	

Color measurements and ∆E* calculations revealed that all three variants, thickness, composites, and opaquer, showed significant effects on the ΔE*. The samples with 1mm thickness, the Vitalescence composite, and the Creative Color opaquer A2, had significantly lower ΔE* than the 0.5mm samples, the Gradia composite, and the Pink opaque, respectively. Detailed results are shown in Table 4. The comparison of the number of samples with desirable (∆E*<3.3) and undesirable color differences (∆E*≥3.3) is shown in Table 5. 

**Table 4:** The effects of different variables on ΔE

		Delta E							P*
		Mean	SD	Median	Minimum	Maximum	95.0% Lower CL for Mean	95.0% Upper CL for Mean	
Thickness	0.5 mm	4.19	2.92	3.42	.42	8.93	3.20	5.18	0.004
	1 mm	2.53	1.63	2.42	.46	5.47	1.98	3.09	
Composite	Vitalescence	2.40	1.74	2.30	.42	5.91	1.81	2.99	0.001
	Gradia	4.33	2.77	4.00	.76	8.93	3.39	5.26	
Opaquer	Pink	5.22	2.14	4.98	2.21	8.93	4.49	5.94	0.001
	Creative A2	1.51	.97	1.09	.42	3.60	1.18	1.84	

**Table 5:** The comparison of samples with desirable and undesirable ΔE

		Delt E status				p *
		Undesirable		Desirable		
		Count	Row N%	Count	Row N%	
Thickness	0.5 mm	18	50.0%	18	50.0%	0.053
	1 mm	10	27.8%	26	72.2%	
Composite	Vitalescence	9	25.0%	27	75.0%	0.016
	Gradia	19	52.8%	17	47.2%	
Opaquer	Pink	27	75.0%	9	25.0%	0.001
	Creative opaquer A2	1	2.8%	35	97.2%	
Restorative Materials	Vitalescence 0.5 mm pink opaquer	8	88.9%	1	11.1%	0.001
	Vitalescence 1 mm pink opaquer	1	11.1%	8	88.9%	
	Gradia 0.5 mm Pink opaquer	9	100.0%	.0	0.0%	
	Gradia 1 mm Pink opaquer	9	100.0%	.0	0.0%	
	Gradia 1 mm Creative opaquer A2	.0	0.0%	9	100.0%	
	Vitalescence 1 mm Creative opaquer A2	.0	0.0%	9	100.0%	
	Vitalescence 0.5mm Creative opaquer A2	.0	0.0%	9	100.0%	
	Gradia 0.5 mm Creative opaquer A2	1	11.1%	8	88.9%	

The type of composite and opaquer was influential in the amount of desirable and undesirable ΔE*. The number of samples with desirable ΔE was significantly higher in the Vitalescence composite group than in the Gradia composite, and the Creative Color A2 opaquer had significantly more desirable ΔE than the Pink opaquer. According to the data, a borderline significant difference in the ΔE* was found between the samples with 1mm thickness and those with 0.5 mm thickness (Table 5). 

## Discussion

In the present study, we used a combination of two opaquers and two resin composites in two thicknesses (0.5 and 1mm) to mask C4 discoloration. Most combinations exhibited acceptable masking ability except those composed of pink opaquer+Vitalescence (0.5mm- thick)/ Gradia (0.5 and 1mm- thick). The null hypothesis was rejected because all three variants influenced the masking ability of the dark background, the resin composite's brand and thickness, and the opaquer's color. 

As in previous studies, we created a 4mm C4 porcelain background, which was used as a standard simulation of the discolored tissue [ 5 , 15 , 17 ].

The inherent color of each composite was taken from a 4-mm-thick disk made from each composite, and it was considered a criterion for comparing the groups with it . The 4-mm thickness was selected because it has been shown that the composite background has no effect on the color of the sample at this thickness [ 18 ].

Previous studies reported different values for clinically significant color difference (∆E*) from 1.7 to 3.7 . According to Mioti *et al*. [ 6 ], B. G. Perez *et al*. [ 10 ], Kim *et al*. [ 5 ], and An *et al*. [ 4 ], a ΔE of equal or higher than 3.3 was considered distinguishable from the clinical point of view. 

To mask a C4 porcelain background, about 0.5mm [ 11 ], 0.5 to 1.0mm [ 5 ], and 0.8 to 1.4mm [ 4 ] of the opaque composites are needed. Therefore, we prepared the samples with 0.5 or 1-mm thicknesses of universal A2 shade resin composite and two layers of opaquer.

In the current study, Vitalescence (Ultradent, USA) and Gradia Direct (GC, Japan) were used. Their selection was based on their availability and ability to mask tooth discoloration [ 4 , 20 - 24 ].

Thickness, as one of the variables, showed a significant effect on masking ability. Samples with higher thicknesses showed significantly higher masking capability; this result is in accordance with previous studies [ 4 , 20 , 25 - 26 ].

The type of composite significantly affected the masking capability, so the Vitalescence composite showed better performance than the Gradia composite. According to previous studies, an increase in the content and size of fillers and irregularity in the shape of fillers lead to a decrease in translucency [ 27 ]. Also, regarding the matrix composition, the Bis-GMA composites exhibited higher translucency values than the UDMA and TE-GDMA [ 28 - 29 ]. The better performance of the Vitalescence composite cannot be justified with the above results due to the presence of some BIS-GMA, slightly smaller size, and lower filler content than Gradia (Table 1).

In line with our results, in the study of Hirata *et al*. [ 30 ], the composite with higher filler loading showed higher translucency. More information is needed on how different factors affect the optical properties of composite resins, including the details of their composition and components. 

In line with our findings, previous studies reported a translucency parameter (TP) of 16.9 for the Gradia composite [ 24 ] and 5.7 and 6.2 for the Vitalescence composite [ 18 , 21 ]. The higher TP values for Gradia show its reduced capability to effectively mask the discolored background. 

In the present study, the highest effect on the color difference was because of the opaquers. The Creative Color opaquer A2 showed lower ∆E* than Pink opaque. In three out of four groups with Pink opaque, the ∆E* was undesirable, while all groups with the Creative Color opaquer A2 had desirable ∆E*. These findings do not agree with the manufacturer’s claim stating that Pink opaque (Cosmedent) has a high capability in masking discolorations, especially over Grey tone (C4 background). One explanation is that, although the pink opaquer may have a strong masking capability, the resulting color is far away from the inherent color of the overlaying composite because of the resulting high value and prominent color in the final restoration. Opaquers with high opacity do not always provide the best results. Excessive opacity may negatively affect the final color of the restoration [ 12 - 13 ]. The use of white opaquers increases the value too much and makes the appearance of the final restoration lifeless [ 1 ]. According to the manufacturer's information, there is no difference in the chemical components of the used opaquers in the present study (Table 1). However, the better performance of Creative Color opaquer A2 in the present study was in line with the positive findings of case report studies regarding the selection of opaquer color as close as possible to the color of final restoration [ 12 , 14 ]. 

In a study by Lehr *et al*. [ 15 ], Creative Color opaquer exhibited positive results in masking discolored backgrounds despite having higher visual translucency than the other opaques. However, further research is needed to compare the optical properties of the opaques.

Nonetheless, the present study had some limitations, including using only one discolored background, not applying different shades of composite by layering technique, and not considering the effect of the optical properties of the opaquer.

Future studies with different combinations of resin shades, layering techniques, and a human observer's visual comparison of the relative translucency between the composites or opaques are recommended. 

## Conclusion

Within the limitations of this study, the following was concluded:

1- The combined use of opaquer and resin composite to mask C4 discoloration was affected by the brand and thickness of the resin composite and the opaque shade.
2- Masking C4 discoloration can be achieved by using two coats of opaquer and 0.5 or 1 mm-thick resin composite, depending on the composite and opaquer used.
3- Most results that achieved acceptable masking ability were obtained with combinations of Creative Color opaquer A2+ Vitalescence/Gradia composite in 0.5 or 1mm thicknesses.
